# 8-Mercaptoguanine-based inhibitors of *Mycobacterium tuberculosis* dihydroneopterin aldolase: synthesis, *in vitro* inhibition and docking studies

**DOI:** 10.1080/14756366.2021.1900157

**Published:** 2021-03-23

**Authors:** Alexia de Matos Czeczot, Candida Deves Roth, Rodrigo Gay Ducati, Kenia Pissinate, Raoní Scheibler Rambo, Luís Fernando Saraiva Macedo Timmers, Bruno Lopes Abbadi, Fernanda Souza Macchi, Víctor Zajaczkowski Pestana, Luiz Augusto Basso, Pablo Machado, Cristiano Valim Bizarro

**Affiliations:** a Instituto Nacional de Ciência e Tecnologia em Tuberculose, Centro de Pesquisas em Biologia Molecular e Funcional, Pontifícia Universidade Católica do Rio Grande do Sul, Porto Alegre, Brazil; b Programa de Pós-Graduação em Biologia Celular e Molecular, Pontifícia Universidade Católica do Rio Grande do Sul, Porto Alegre, Brazil; c Programa de Pós-Graduação em Medicina e Ciências da Saúde, Pontifícia Universidade Católica do Rio Grande do Sul, Porto Alegre, Brazil; d Programa de Pós-Graduação em Biotecnologia, Universidade do Vale do Taquari, Lajeado, Brazil

**Keywords:** Tuberculosis, dihydroneopterin aldolase, 8-mercaptoguanine, MtDHNA/MtFolB inhibition

## Abstract

The dihydroneopterin aldolase (DHNA, EC 4.1.2.25) activity of FolB protein is required for the conversion of 7,8-dihydroneopterin (DHNP) to 6-hydroxymethyl-7,8-dihydropterin (HP) and glycolaldehyde (GA) in the folate pathway. FolB protein from *Mycobacterium tuberculosis* (*Mt*FolB) is essential for bacilli survival and represents an important molecular target for drug development. S8-functionalized 8-mercaptoguanine derivatives were synthesised and evaluated for inhibitory activity against *Mt*FolB. The compounds showed IC_50_ values in the submicromolar range. The inhibition mode and inhibition constants were determined for compounds that exhibited the strongest inhibition. Additionally, molecular docking analyses were performed to suggest enzyme-inhibitor interactions and ligand conformations. To the best of our knowledge, this study describes the first class of *Mt*FolB inhibitors.

## Introduction

Tuberculosis (TB) is one of the oldest diseases that remain a health concern worldwide due to high incidence and mortality rates. According to the World Health Organisation (WHO), an estimated 10 million people fell ill in 2019, while 1.4 million people died with TB in the same period^1^. Duration, complexity of treatment, and drug side effects result in poor adherence, suboptimal response, treatment failure, emergence of drug resistance, and continuous disease spread. Therefore, new and more effective treatments are urgently needed^2^^,^^3^.

Folate and its derivatives act as cofactors in the biosynthesis of purines, pyrimidines, and amino acids^4^. Antifolates interrupt the production of folate and its derivatives by inhibiting key enzymes in the folate metabolic pathway^5^. Among the enzymes of this pathway, only dihydropteroate synthase (DHPS) and dihydrofolate reductase (DHFR) are currently used as targets for antimicrobial agents^6^. Despite the antimycobacterial activity of antifolates in culture and the use of *para*-aminosalicylic acid (PAS) as a second-line drug, these molecules are not used in the first-line treatment of TB^7^^,^^8^. The FolB protein, encoded by the *folB* gene, is a dihydroneopterin aldolase enzyme (DHNA, EC 4.1.2.25), as it converts 7,8-dihydroneopterin (DHNP) to 6-hydroxymethyl-7,8-dihydropterin (HP) and glycolaldehyde (GA) in the third step of the folate pathway. FolB from *Mycobacterium tuberculosis* (*Mt*FolB) is also a dihydromonapterin (DHMP) aldolase, converting DHMP to HP and GA, an epimerase, interconverting DHNP and DHMP, and an oxygenase, producing 7,8-dihydroxantopterin (DHXP) from either DHNP or DHMP^9^. This protein is the first of the three enzymes from the folate pathway that are absent in mammals and represents an attractive target for the development of antimicrobial agents^4^.

**Scheme 1.**
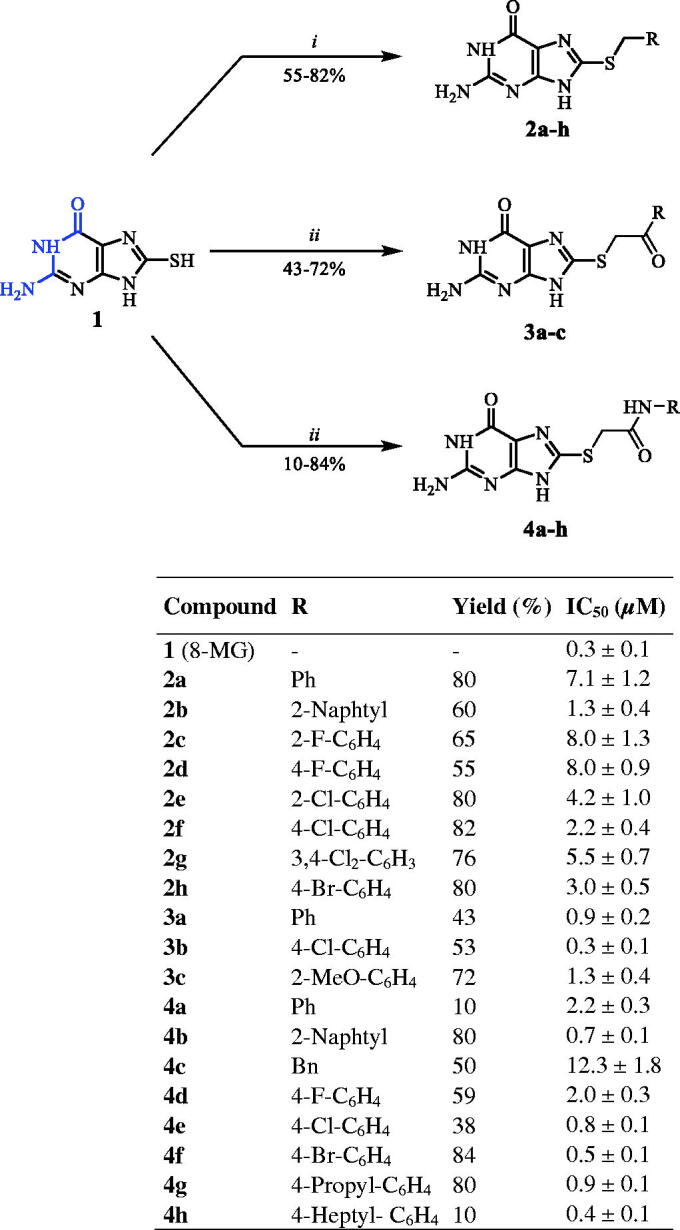
Reagents and conditions. (*i*) = Benzyl bromide, NaOH, EtOH, 25 °C, 4 h. (*ii*) 2-bromo-1-arylethanone or bromoacetamide, NaOH, EtOH, 25 °C, 24 h.

We have shown previously that the *folB* gene from *M. tuberculosis* is essential for bacilli survival under defined conditions and that its essentiality depends on the aldolase and/or epimerase activities of *Mt*FolB protein^10^. This paved the way for the development of *Mt*FolB aldolase/epimerase inhibitors as potential anti-TB agents. Compounds with inhibitory activity against the orthologous enzyme from *Staphylococcus aureus* (*Sa*FolB) were previously identified in a high-throughput X-ray crystallographic screening using an initial library with 10 000 compounds^11^. In this same study, a new sublibrary of approximately 1 000 compounds was constructed, all containing the H_2_N-C-NH-C=O substructure in common (highlighted in blue in Scheme 1). Several hit compounds with low IC_50_ values against *Sa*FolB were identified, including 8-mercaptoguanine (8-MG), with an IC_50_ value of 1 µM. X-ray crystallography revealed that the H_2_N-C-NH-C=O substructure shared by 8-MG, the substrate analogue neopterin and also the product HP presented hydrogen bonds with the same *Sa*FolB residues in these three ligand-protein complexes^11^. Furthermore, 8-MG inhibits 6-hydroxymethyl-7,8-dihydropterin pyrophosphokinase from *S. aureus* (*Sa*HPPK), another enzyme of the folate pathway, with an IC_50_ value of 41 µM^12^. Structure-activity relationship (SAR) studies have been performed to identify structural analogues of 8-MG with greater potential to inhibit enzymes from the folate biosynthesis pathway. S8-functionalized derivatives of 8-MG with improved affinity for both *Sa*HPPK and *Escherichia coli* HPPK (*Ec*HPPK) were reported^13^^,^^14^. Additionally, novel S8-functionalized derivatives of 8-MG with submicromolar affinities for another folate enzyme,dihydropteroate synthase (DHPS), from *E. coli* (EcDHPS) were described and structurally characterized^15^.

In this study, we investigate the inhibitory potential of 8-MG and S8-functionalized 8-MG derivatives against *Mt*FolB. We synthesised 19 S8-functionalized compounds using 8-MG as a scaffold and evaluated their inhibitory activity *in vitro* against *Mt*FolB enzyme. In this way, we identified new inhibitors for *Mt*FolB and provided the mode of inhibition of the top hits. We simulated the enzyme-inhibitor interactions by molecular docking and evaluated the growth inhibitory activities for Mtb *in vitro* for 8-MG and the derivatives reported here.

## Materials and methods

### Chemical synthesis

Reagents, chemicals, starting materials and solvents were obtained from commercial sources and used without further purification. Melting points were determined on an Microquímica MQAPF-302 apparatus. IR spectra were recorded on Perkin-Elmer Spectrum 100 FT-IR spectrometer with a Universal ATR sampling accessory. NMR spectra were recorded on a Avance III HD Bruker spectrometer with chemical shifts values (*δ*) in ppm relative to TMS using the residual DMSO-*d6* signal as an internal standard. High-resolution mass spectra (HRMS) were recorded on an LTQ Orbitrap Discovery mass spectrometer (Thermo Fisher Scientific, Bremen, Germany). This system combines an LTQ XL linear ion-trap mass spectrometer and an Orbitrap mass analyser. The analyses were performed by direct infusion of the sample in MeOH/CH_3_CN (1:1) with 0.1% formic acid (flow rate of 10 µL/min) in positive-ion mode using electrospray ionisation (ESI). For the elemental composition, the calculations used the specific tool included in the Qual Browser module of Xcalibur (Thermo Fisher Scientific, release 2.0.7) software.

### General procedure for synthesis of compounds 2a–h

Compounds **2a**–**h** were synthesised as previously described^13^. In brief, 8-mercaptoguanine (scaffold molecule **1**−8-MG) (0.2 g, 1.09 mmol) was dissolved in 0.5 M NaOH (5.5 ml) resulting in a real solution. To this solution was added the respective benzyl bromide (1.2 mmol) in ethanol (0.9 ml). The reaction was stirred for 4h at 25 °C and the precipitated formed was collected by vacuum filtration affording the title compound as a white-yellowish amorphous solid.

#### 2-Amino-8-(benzylthio)-1,9-dihydro-6H-purin-6-one (2a)

80% yield. MP = 270–273 °C. ^1^H NMR (400 MHz, DMSO-*d*_6_) *δ*: 12.55 (bs, 1H), 10.63 (bs, 1H), 7.47–7.20 (m, 5H), 6.34 (bms, 2H), 4.40 (d, *J* = 4.5 Hz, 2H). IR-ATR (cm^−1^): 3321, 3086, 1651. HRMS (ESI): calc. for [C_12_H_11_N_5_OS + H]^+^: 274.0757; obt.: 274.0748.^13^

#### 2-Amino-8-((naphthalen-2-ylmethyl)thio)-1,9-dihydro-6H-purin-6-one (2b)

60% yield. MP = 286–287 °C. ^1^H NMR (400 MHz, DMSO-*d*_6_) *δ*: 12.59 (bs, 1H), 10.59 (bs, 1H), 7.99–7.78 (m, 4H), 7.63–7.39 (m, 3H), 6.31 (bs, 2H), 4.58 (s, 2H). IR-ATR (cm^−1^): 3325, 3103, 2869, 1671, 1653. HRMS (ESI): calc. for [C_16_H_13_N_5_OS + H]^+^: 324.0914; obt.: 324.0910.^15^

#### 2-Amino-8-((2-fluorobenzyl)thio)-1,9-dihydro-6H-purin-6-one (2c)

65% yield. MP = 289–290 °C. ^1^H NMR (400 MHz, DMSO-*d*_6_) *δ*: 12.57 (bs, 1H), 10.57 (bs, 1H), 7.50–7.27 (m, 2H), 7.24–7.07 (m, 2H), 6.36 (s, 2H), 4.38 (s, 2H). IR-ATR (cm^−1^): 3324, 3102, 2879, 1673, 1651. HRMS (ESI): calc. for [C_12_H_10_FN_5_OS + H]^+^: 292.0663; obt.: 292.0652.^13^

#### 2-Amino-8-((4-fluorobenzyl)thio)-1,9-dihydro-6H-purin-6-one (2d)

55% yield. MP = 299–300 °C. ^1^H NMR (400 MHz, DMSO-*d*_6_) *δ*: 12.53 (bs, 1H), 10.58 (bs, 1H), 7.41 (t, *J* = 6.9 Hz, 2H), 7.21–7.05 (m, 2H), 6.34 (bs, 1H), 4.38 (s, 2H). IR-ATR (cm^−1^) 3324, 3103, 2860, 1652. HRMS (ESI): calc. for [C_12_H_10_FN_5_OS + H]^+^: 292.0663; obt.: 292.0670.^14^

#### 2-Amino-8-((2-chlorobenzyl)thio)-1,9-dihydro-6H-purin-6-one (2e)

80% yield. MP = 301–302 °C. ^1^H NMR (400 MHz, DMSO-*d*_6_) *δ*: 12.53 (bs, 1H), 10.54 (bs, 1H), 7.53–7.42 (m, 2H), 7.28 (m, 2H), 6.24 (s, 2H), 4.48 (s, 2H). IR-ATR (cm^−1^): 3310, 3101, 2880, 1659. HRMS (ESI): calc. for [C_12_H_10_ClN_5_OS + H]^+^: 308.0367; obt.: 308.0358.^14^

#### 2-Amino-8-((4-chlorobenzyl)thio)-1,9-dihydro-6H-purin-6-one (2f)

82% yield. MP = 291–294 °C. ^1^H NMR (400 MHz, DMSO-*d*_6_) *δ*: 12.55 (bs, 1H), 10.63 (bs, 1H), 7.38 (d, *J* = 10.2 Hz, 4H), 6.32 (bs, 2H), 4.38 (s, 2H). IR-ATR (cm^−1^): 3328, 3097, 2865, 1653. HRMS (ESI): calc. for [C_12_H_10_ClN_5_OS + H]^+^: 308.0367; obt.: 308.0376.^14^

#### 2-Amino-8-((2,4-dichlorobenzyl)thio)-1,9-dihydro-6H-purin-6-one (2g)

76% yield. MP = 290–291 °C. ^1^H NMR (400 MHz, DMSO-*d*_6_) *δ*: 10.64 (bs, 1H), 7.63 (s, 1H), 7.54 (d, *J* = 8.2 Hz, 1H), 7.33 (d, *J* = 8.2 Hz, 1H), 6.33 (bs, 2H), 4.37 (s, 2H). IR-ATR (cm^−1^): 3413, 3276, 3146, 1677. HRMS (ESI): calc. for [C_12_H_9_Cl_2_N_5_OS + H]^+^: 341.9978; obt.: 341.9971.

#### 2-Amino-8-((4-bromobenzyl)thio)-1,9-dihydro-6H-purin-6-one (2h)

80% yield. MP = 301–302 °C. ^1^H NMR (400 MHz, DMSO-*d*_6_) *δ*: 12.53 (bs, 1H), 10.60 (d, *J* = 41.2 Hz, 1H), 7.61–7.21 (m, 4H), 6.25 (d, *J* = 74.4 Hz, 2H), 4.38 (d, *J* = 27.6 Hz, 2H). IR-ATR (cm^−1^): 3328, 3173, 3098, 2864, 1653. HRMS (ESI): calc. for [C_12_H_10_BrN_5_OS + H]^+^: 351.9862; Obt.: 351.9861.^14^

### General procedure for synthesis of compounds 3a–c and 4a–h

Compounds **3a**–**c** and **4a**–**h** were synthesised according to an already reported protocol with minor modifications^13^. In brief, 8-MG (**1**) (0.2 g, 1.09 mmol) was dissolved in 0.5 M NaOH (5.5 ml), and to the resulting solution was added the respective bromoacetamide or 2-bromoacetophenone (1.2 mmol) in ethanol (0.9 ml). The reaction was stirred for 24h at 25 °C, then 1% acetic acid was added until pH = 5. The mixture was extracted with ethyl acetate, the organic layers were combined, dried with MgSO_4_ and evaporated under reduced pressure. The residue was purified by *flash* column chromatography eluting a mixture of chloroform and methanol (9:1 → 1:1) or recrystallized in methanol to give the title compound as a white-yellowish amorphous solid.

#### 2-Amino-8-((2-oxo-2-phenylethyl)thio)-1,9-dihydro-6H-purin-6-one (3a)

31% yield. MP > 310 °C. ^1^H NMR (400 MHz, DMSO-*d*_6_) *δ*: 12.51 (bs, 1H), 10.55 (bs 1H), 8.20–7.26 (m, 2H), 7.78–7.47 (m, 3H), 6.29 (bs, 2H), 4.85 (s, 2H). IR-ATR (cm^−1^): 3322, 3098, 2919, 1651. HRMS (ESI): calc. for [C_13_H_11_N_5_O_2_S + H]^+^: 302.0706; obt.: 302.0706.^13^

#### 2-Amino-8-((2–(4-chlorophenyl)-2-oxoethyl)thio)-1,9-dihydro-6H-purin-6-one (3b)

53% yield. MP = 300–316 °C. ^1^H NMR (400 MHz, DMSO-*d*_6_) *δ*: 12.53 (bs, 1H), 10.82 (bs, 1H), 8.04 (d, *J* = 8.3 Hz, 2H), 7.63 (d, *J* = 8.2 Hz, 2H), 6.52 (bs, 2H), 4.88 (d, *J* = 37.0 Hz, 2H). IR-ATR (cm^−1^): 3094, 1673, 1586. HRMS (ESI): calc. for [C_13_H_10_ClN_5_O_2_S + H]^+^: 336.0316; obt.: 336.0325.^14^

#### 2-Amino-8-((2–(2-methoxyphenyl)-2-oxoethyl)thio)-1,9-dihydro-6H-purin-6-one (3c)

72% yield. MP = 309–311 °C. ^1^H NMR (400 MHz, DMSO-*d*_6_) *δ*: 12.51 (bs, 1H), 10.54 (bs, 1H), 7.76–7.50 (m, 2H), 7.21 (d, *J* = 8.5 Hz, 1H), 7.05 (td, *J* = 7.5, 1.0 Hz, 1H), 6.35 (bs, 2H), 4.71 (s, 2H), 3.93 (s, 3H). IR-ATR (cm^−1^): 3322, 3097, 1673, 1652. HRMS (ESI): calc. for [C_14_H_13_N_5_O_3_S + H]^+^: 332.0812; obt.: 332.0820.^13^

#### 2-((2-Amino-6-oxo-6,9-dihydro-1H-purin-8-yl)thio)-N-phenylacetamide (4a)

10% yield. MP = 291–293 °C. ^1^H NMR (400 MHz, DMSO-*d*_6_) *δ*: 10.53 (bs, 2H), 7.57 (d, *J* = 8.0 Hz, 2H), 7.30 (t, *J* = 7.9 Hz, 2H), 7.05 (t, *J* = 7.4 Hz, 1H), 6.32 (bs, 2H), 4.07 (s, 2H). IR-ATR (cm^−1^): 3321, 3145, 3070, 1665. HRMS (ESI): calc. for [C_13_H_12_N_6_O_2_S + H]^+^: 317.0815; obt.: 317.0817.^15^

#### 2-((2-Amino-6-oxo-6,9-dihydro-1H-purin-8-yl)thio)-N-(naphthalen-2-yl)acetamide (4b)

80% yield. MP = 286–289 °C. ^1^H NMR (400 MHz, DMSO-*d*_6_) *δ*: 11.01 (bs, 1H), 8.31 (s, 1H), 7.93–7.75 (m, 3H), 7.60 (dd, *J* = 8.8, 2.1 Hz, 1H), 7.44 (dddd, *J* = 27.2, 8.1, 6.8, 1.3 Hz, 2H), 7.36–7.13 (m, 1H), 6.34 (bs, 2H), 4.13 (s, 2H). IR-ATR (cm^−1^): 3320, 3147, 1666. HRMS (ESI): calc. for [C_17_H_14_N_6_O_2_S + H]^+^: 367.0972; obt.: 367.0964.

#### 2-((2-Amino-6-oxo-6,9-dihydro-1H-purin-8-yl)thio)-N-benzylacetamide (4c)

50% yield. MP = 270–271 °C. 1H NMR (400 MHz, DMSO-*d*_6_) *δ*: 12.54 (s, 1H), 10.55 (bs, 1H), 8.71 (s, 1H), 7.33–7.17 (m, 5H), 6.39–6.32 (m, 2H), 4.30 (d, *J* = 6.0 Hz, 3H), 3.92 (s, 2H). IR-ATR (cm^−1^): 3303, 3164, 2987, 2879, 1663. HRMS (ESI): calc. for [C_14_H_14_N_6_O_2_S + H]^+^: 331.0972; obt.: 331.0966.

#### 2-((2-Amino-6-oxo-6,9-dihydro-1H-purin-8-yl)thio)-N-(4-fluorophenyl)acetamide (4d)

59% yield. MP = 243–245 °C. ^1^H NMR (400 MHz, DMSO-*d*_6_) δ: 11.08 (bs, 1H), 7.76–7.54 (m, 2H), 7.27–7.08 (m, 2H), 6.39 (bs, 1H), 4.01 (d, *J* = 2.9 Hz, 2H). IR-ATR (cm^−1^): 3073, 1615, 1507. HRMS (ESI): calc. for [C_13_H_11_FN_6_O_2_S + H]^+^: 335.0721; obt.: 335.0726.

#### 2-((2-Amino-6-oxo-6,9-dihydro-1H-purin-8-yl)thio)-N-(4-chlorophenyl)acetamide (4e)

38% yield. MP = 306–308 °C. ^1^H NMR (400 MHz, DMSO-*d*_6_) *δ*: 12.49 (bs, 1H), 10.69 (s, 1H), 7.72–7.53 (m, 2H), 7.46–7.30 (m, 2H), 6.71–6.55 (m, 2H), 4.10 (s, 2H). IR-ATR (cm^−1^): 3073, 1615, 1507. HRMS (ESI): calc. for [C_13_H_11_ClN_6_O_2_S + H]^+^: 351.0425; obt.: 351.0428.

#### 2-((2-Amino-6-oxo-6,9-dihydro-1H-purin-8-yl)thio)-N-(4-bromophenyl)acetamide (4f)

84% yield. MP > 310 °C. 1H NMR (400 MHz, DMSO-*d*_6_) *δ*: 10.75 (bs, 1H), 7.62–7.54 (m, 2H), 7.53–7.46 (m, 2H), 6.37 (bs, 2H), 4.09 (s, 2H). IR-ATR (cm^−1^): 3310, 3124, 1668. HRMS (ESI): calc. for [C_13_H_11_BrN_6_O_2_S + H]^+^: 394.9912; obt.: 394.9920.

#### 2-((2-Amino-6-oxo-6,9-dihydro-1H-purin-8-yl)thio)-N-(4-propylphenyl)acetamide (4g)

80% yield. MP = 299–301 °C. ^1^H NMR (400 MHz, DMSO-*d*_6_) δ: 10.41 (bs, 1H), 7.48 (d, *J* = 8.1 Hz, 2H), 7.11 (d, *J* = 8.4 Hz, 2H), 6.48 (bs, 3H), 4.09 (s, 2H), 2.55–2.41 (m, 2H), 1.53 (p, *J* = 7.3 Hz, 2H), 0.86 (t, *J* = 7.3 Hz, 3H). IR-ATR (cm^−1^): 2927, 1669, 1600, 1513. HRMS (ESI): Calc. for [C_16_H_18_N_6_O_2_S + H]^+^: 359.1285; obt.: 359.1287.

#### 2-((2-Amino-6-oxo-6,9-dihydro-1H-purin-8-yl)thio)-N-(4-heptylphenyl)acetamide (4h)

80% yield. MP > 310 °C. ^1^H NMR (400 MHz, DMSO-*d*_6_) *δ*: 10.83 (bs, 1H), 7.59–7.39 (m, 2H), 7.27–7.03 (m, 2H), 6.53 (bs, 2H), 4.32–3.99 (m, 2H), 1.52 (s, 2H), 1.24 (m, *J* = 9.5 Hz, 10H), 0.99–0.70 (m, 3H). IR-ATR (cm^−1^): 3291, 3075, 2923, 2851, 1673, 1610. HRMS (ESI): Calc. for [C_20_H_26_N_6_O_2_S + H]^+^: 415.1911; obt.: 415.1909.

### Expression, purification, and continuous fluorescence-based enzyme activity assay

The expression and purification of recombinant *Mt*FolB were performed as previously described^10^. A continuous fluorescence-based enzyme activity assay was optimized^9^ for monitoring the aldolase reaction of *Mt*FolB (conversion of DHNP to HP and GA) by an increase in fluorescence due to HP formation on an RF-5301 spectrofluorophotometer (Shimadzu) with an excitation wavelength of 365 nm and fluorescence emission at 525 nm. The slits were 10 and 15 nm for excitation and emission, respectively. To determine the apparent steady-state kinetic constants, *Mt*FolB activity was monitored at varying concentrations of DHNP (0.10 − 10 µM) using 300 nM *Mt*FolB in 25 mM Tris, 50 mM NaCl, 5% glycerol pH 8.0 at 25 °C for 6 min in a final volume of 1.0 ml. Control reactions (buffer only, buffer + substrate, buffer + enzyme) were performed under the same conditions to subtract fluorescence intensities not coming from the reaction product. The data were fitted to Equation (1) for a saturation curve, in which *v* is initial velocity, *V*_max_ is the maximum velocity, *E*_0_ is the initial total enzyme concentration, *S*_T_ is the initial total substrate concentration, and *K*_M_ is the Michaelis − Menten constant for the substrate used^9^. A calibration curve ranging from 0.020 to 15 µM of HP was performed. The slope of the fluorescence emission at 525 nm as a function of HP concentration was applied to obtain the catalytic constant (*k*_cat_) values for the aldolase reactions:

(1) $$v=V_{\text{max }}\times \;\frac{(E_{0}+\;S_{T}+\;K_{M})-\;\sqrt{{\left(E_{0}+\;S_{T}+\;K_{M}\right)}^{2}-4\;\times \;E_{0}\;\times \;S_{T}}}{2\;\times \;E_{0}}$$

### Enzyme inhibition assays

Enzyme inhibition studies were performed using an RF-5301 spectrofluorophotometer (Shimadzu), monitoring an increase in fluorescence at 525 nm for HP formation for 6 min. The presence of time-dependent inhibitory activity was evaluated for 8-MG (**1**) and the compounds synthesised. For this analysis, 300 nM (final concentration) of recombinant *Mt*FolB was preincubated with a fixed inhibitor concentration defined for each compound (final concentrations of 360 nM for 8-MG (**1**); 500 nM for **3b** and **4h**; 625 nM for **4g**, **4f** and **4e**; 830 nM for **4b** and **4d**; 1.0 µM for **3a**; 1.25 µM for **3c**, **2g** and **2f**; 1.65 µM for **2b** and **2h**; 2.5 µM for **4c**; 5 µM for **2e** and **4a**; or 7 µM for **2d**, **2c** and **2a**), which was then added at different times (up to 40 min) to the reaction mixture (DHNP at *K*_M_ value, 25 mM Tris, 50 mM NaCl, 5% glycerol pH 8.0 and final concentration of 2% DMSO). The change in initial velocity as a function of time was monitored and the percentage of inhibition was calculated. As a control, *Mt*FolB was preincubated with DMSO alone at a maximum final concentration of 2% and added to the reaction mixture. All experiments were performed at 25 °C.

The IC_50_ values for the compounds were determined in the reaction conditions aforementioned. We fixed DHNP at a non-saturating concentration (∼ *K*_M_ value) and dissolved the compounds in DMSO at varied concentrations (8-MG (**1**): 0.1 − 1.0 µM; **3b** and **4h**: 0.1 − 1.2 µM; **4b**: 0.2 − 3.0 µM; **4e** and **4g**: 0.2 − 2.5 µM; **4f**: 0.2 − 2.0 µM; **3a**: 0.35 − 5 µM; **4d**: 0.4 − 4.0 µM; **4a**: 0.5 − 10.0 µM; **3c**: 0.6 − 3.0 µM; **2f**: 1.0 − 7.0 µM; **2g**: 1.0 − 15 µM; **2b**: 0.6 − 5.0 µM; **2h**: 1.5 − 15 µM; **4c**: 3.0 − 40 µM; **2d**, **2c** and **2a** 2.0 − 30 µM; **2e** 2.0 − 15 µM). The maximal rate of the enzymatic reaction (100% of *Mt*FolB activity) was determined with 2% DMSO in the absence of inhibitor. IC_50_ values were estimated using Equation (2), where *V*_i_ and *V*_0_ are, respectively, the reaction velocity in the presence and in the absence of inhibitor (I):

(2) $$\frac{V_{i}}{V_{0}}\;=\;\frac{1}{1+\frac{\left[I\right]}{{\mathrm{IC}}_{50}}}$$

The determination of the mode of inhibition (competitive, non-competitive, or uncompetitive) and the inhibition constants (*K*_is_ and/or *K*_ii_) were performed for each selected inhibitor with an IC_50_ value < 0.50 µM. We consider as competitive inhibitors compounds that bind only the free enzyme, as non-competitive inhibitors the ones that bind both the enzyme-substrate complex and the free enzyme (but not necessarily with the same binding affinity), and uncompetitive inhibitors the compounds that bind exclusively the enzyme-substrate complex^17^.

The inhibition studies were carried out at varying concentrations of DHNP until saturation, and fixed-varied inhibitor concentrations. For 8-MG (**1**) the fixed-varied concentrations were 0.15 µM, 0.30 µM (varying DHNP 0.25 − 10 µM) and 0.45 µM (varying DHNP 0.35 − 10 µM). For **3b** the fixed-varied concentrations were 0.20 µM (varying DHNP 0.35 − 10 µM), 0.40 µM (varying DHNP 0.35 − 15 µM), and 0.80 µM (varying DHNP 0.5 − 20 µM). For **4f** the fixed-varied concentrations were 0.30 µM (varying DHNP 0.35 − 10 µM), 0.50 µM and 0.70 µM (varying DHNP 0.5 − 15 µM). For **4h** the fixed-varied concentrations were 0.30 µM, 0.50 µM (varying DHNP 0.35 − 10 µM), and 0.70 µM (varying DHNP 0.5 − 15 µM). The enzyme concentration was constant at 300 nM throughout the assays. The mode of inhibition of compounds was determined from the straight-line patterns, and *K*_is_ and/or *K*_ii_ values towards DHNP were estimated using Equation (3) or Equation (4), which describe a non-competitive and competitive inhibition, respectively. Data were fitted to the following equations:

(3) $$v_{0}=\frac{V_{\max}\;[S]}{K_{M}\;(\;1+\;\frac{\left[I\right]}{K_{\mathrm{is}}}\;)\;+\;[S]\;(1+\;\frac{\left[I\right]}{K_{\mathrm{ii}}})}$$

(4) $$v_{0}=\;\frac{V_{\max}\;[S]}{\left[S]\;+\;K_{M}\;(\;1+\;\frac{\left[I\right]}{K_{\mathrm{is}}}\;\right)}$$

where [I] is the inhibitor concentration, [S] is the substrate concentration, *K*_M_ and *V*_max_ are the Michaelis − Menten constant and maximum velocity, respectively, *K*_ii_ is the overall inhibition constant for the enzyme − substrate − inhibitor complex and *K*_is_ is the overall inhibition constant for the enzyme − inhibitor complex^16^.

### Molecular docking protocol

Molecular docking simulations were carried out to evaluate the orientation and binding affinity of 8-MG (**1**) and its derivatives into the binding pocket of *Mt*FolB. Prior to this, we performed a redocking procedure, using the crystallographic structures of *Mt*FolB as an octamer (PDB ID 1NBU)^17^ aiming to verify whether our protocol could reproduce the ligand location found in the experimental structure. The flexible docking simulations were performed using PyrX-0.9.4^18^, where the AutoDock 4.2 isimplemented^19^. The AutoDock software uses an empirical scoring function based on the free energy of binding. Among the stochastic search algorithms offered by the AutoDock suite, we chose the Lamarckian genetic algorithm (LGA) that is a hybrid approach, which combines genetic algorithm (as global search)^20^ and Solis and West algorithm (as local search)^21^.

A grid box was created with 50 × 50 × 50 points and a resolution of 0.375 Å to include solely the protein’s active site to reduce the computational cost. The coordinates of the grid centre was *x* − 1.79, *y* − 23.47, and *z* 21.63. The molecular docking process was carried out with 60 independent runs for each docking simulation, an initial population of 400, a maximum number of 4000 000 energy evaluation, and a maximum number of 27 000 generations. Mutation and crossover were applied to the population at rates 0.02 and 0.80, respectively.

### *Mycobacterium tuberculosis* inhibition assay

The inhibitory potential of the compounds was evaluated against *M. tuberculosis* H37Rv reference strain (ATCC 27294) by the resazurin reduction microplate assay (REMA) as previously described^22^. Stock solutions (0.5 mg mL^−1^ for 8-MG (**1**) and 2 mg mL^−1^ for all other test compounds) were made in neat DMSO (Sigma-Aldrich) and aliquots were stored at −20 °C. The assays were performed in Difco™ Middlebrook 7H9 broth (Becton Dickinson – BD) supplemented with 10% (v/v) BBL™ Middlebrook ADC enrichment (albumin, dextrose and catalase – BD) and 2.5% (v/v) DMSO. The maximum concentration tested varied among compounds due to differences in solubility (2.5 − 40 µg mL^−1^). The minimal inhibitory concentration (MIC) was determined by performing 10-point 2-fold serial dilutions for each compound. Three independent experiments were performed, and MIC was considered as the lowest compound concentration that prevented the resazurin (Sigma-Aldrich) colour conversion from blue (inhibition) to pink (growth). The MIC values stated for the compounds were the most frequent values among the three experiments, or the highest value observed.

## Results and discussion

The synthesis of compounds **2a-h**, **3a-c**, and **4a-h** was accomplished through *S*-alkylation in a nucleophilic substitution reaction. Our strategy was to attach hydrophobic side chains to 8-mercaptoguanine (**1**) to obtain enzymatic inhibitors with physicochemical properties that could facilitate the permeability of the molecules and increase the chance of obtaining structures with potent antimycobacterial activity. The alkylating agents were chosen from different aryl(nafthyl) groups containing electron-donating and electron-withdrawing groups as substituents.

The dihydro-purinones **2a**–**h** were obtained from the reaction of 8-mercaptoguanine (**1**) and benzyl bromides in the presence of sodium hydroxide (NaOH) as a base and ethanol (EtOH) as the solvent. The reactants were stirred for 4h at 25 °C, leading to products **2a**–**h** with 55–82% yields (Scheme 1). Using the same conditions for 24h, the compounds **3a**–**c** were synthesised by the reaction of 8-mercaptoguanine (**1**) and 2-bromo-1-arylethanones, with 43 − 72% yields (Scheme 1). Finally, using the same procedure described above, the dihydro-purinones **4a**–**h** were obtained from the reaction of 8-mercaptoguanine (**1**) and bromoacetamides, with 10 − 84% yields (Scheme 1). In general, the presence of a carbonyl group in the alkylating agent provided products in lower yields when compared to the reactions using benzyl bromides.

The synthesised compounds **2a-h, 3a-c, and 4a-h** were evaluated as inhibitors of *Mt*FolB aldolase activity using a continuous fluorescence assay. The Michaelis–Menten constant (*K*_M_) was determined at varying concentrations of DHNP until enzyme saturation (Figure S1, Supplementary Material). *K*_M_ and *k*_cat_ values of 1.42 ± 0.13 µM and 0.011 ± 0.0003s−^1^ were obtained, respectively. The values determined here differ from the values previously reported for this enzyme (*K*_M_ = 0.165 ± 0.026 µM and *k*_cat_ = 0.0054 ± 0.0002s−^1^)^9^. This should be attributed to differences in the method of enzyme purification and the buffer and pH of the enzyme activity assay; changes in solution conditions can affect the apparent value of *K*_M_, influencing the ability of the enzyme to combine with substrate^16^.

The inhibitory potential of 8-mercaptoguanine (8-MG (**1**)) and the synthesised compounds was evaluated against *Mt*FolB. No time dependence was demonstrated up to 40 min of preincubation with *Mt*FolB (data not shown). The initial screening of 20 compounds showed inhibition with IC_50_ values ranging from 0.3 to 12.3 µM (Scheme 1).

Based on IC_50_ values, benzyl-containing compounds **2a**-**h** showed lower activity than 8-MG (**1**) (Scheme 1). The unsubstituted compound **2a** exhibited an IC_50_ of 7.1 µM whereas 8-MG (**1**) has an IC_50_ of 0.3 µM for *Mt*FolB. Compared to the benzyl derivative **2a**, the molecular volume increase with the use of the naphthyl group in the dihydro-purinone **2b** improved the activity more than 5-fold, leading to an IC_50_ of 1.3 µM. By contrast, the presence of a fluorine atom at the 2- (**2c**) or 4- (**2d**) position of the benzene ring led to molecules with reduced activities (IC_50_ = 8.0 µM). Once more, increasing molecular volume with change of fluorine by chlorine atom improved the inhibitory activity towards *Mt*FolB, resulting in an IC_50_ of 4.2 and 2.2 µM for compounds **2e** and **2f**, respectively. Substitution with chlorine atoms at position 3 and 4 of the benzyl ring reduced the inhibitory activity of *Mt*FolB. 3,4-Dichlorophenyl-substituted **2g** showed an IC_50_ of 5.5 µM, which was 2.5-fold higher than its monosubstituted analog, **2f**. Additionally, the 4-bromophenyl-substituted **2h** exhibited an IC_50_ of 3.0 µM, denoting that the classic bioisosteric replacement between the chlorine and bromine was able to maintain similar and reduced potencies.

In the second round, carbonyl-containing compounds **3a-c** were evaluated as inhibitors of *Mt*FolB activity. The presence of this hydrogen bond acceptor group could lead to more potent structures when compared to benzyl derivatives **2a-h**. Indeed, dihydro-purinones **3a-c** were more potent than their counterparts **2a-h**. The phenyl derivative **3a** exhibited an IC_50_ of 0.9 µM. When chlorine atom was positioned at 4-position of the benzene ring in the compound **3b**, the capacity to inhibit the *Mt*FolB was increased. The IC_50_ presented by structure **3b** was 0.3 µM. This IC_50_ value indicated an equipotent activity compared to that presented by 8-MG (**1**). The presence of the methoxy group attached at the 2-position of the benzene ring yielded compound **3c**, which exhibited an IC_50_ of 1.3 µM. This result demonstrates that this electron-donating group reduced in more than 4-fold the inhibitory capacity of this molecule when compared to the activity presented by structure **3b**.

In view of these results, our research focus was directed to the insertion of an amide group to the molecules. If ketone carbonyl groups were responsible for the increase in activity, the presence of a more potent hydrogen bond acceptor (amide) could lead to more potent inhibitors. Such hypothesis started to be evaluated by the unsubstituted derivative **4a** which showed an IC_50_ of 2.2 µM. Similar to that observed with dihydro-purinones **2a-h**, the presence of the naphthyl group significantly increased the inhibitory activity. The compound **4b** exhibited an IC_50_ of 0.7 µM. Interestingly, the use of methylene as a spacer in the **4c** reduced the activity to a great extent. Structure **4c** presented IC_50_ of 12.3 µM which was near 5.6-fold less active than phenyl derivative **4a**. This result denotes that the amide planarity can be crucial for the activity shown by the synthesised compounds. Dihydro-purinone **4d**, containing a fluorine atom at the 4-position of benzene ring, showed an IC_50_ of 2.0 µM. When fluorine atom was changed by 4-chloro, the capacity to inhibit *Mt*FolB increased. The IC_50_ value of the compound **4e** was 0.8 µM which indicated a 2.5-fold increase in the inhibitory activity compared to that exhibited by 4-fluor-substituted structure **4d**. The bromine atom attached at the 4-position of **4f** yielded a molecule with IC_50_ of 0.5 µM. Once more, increasing the volume of the substituent in this portion of the molecule seems to favour its inhibitory activity towards *Mt*FolB. Finally, positioning propyl (**4g**) and heptyl (**4h**) groups at position of the benzene ring led to structures with IC_50_ of 0.9 and 0.4 µM, respectively. Interestingly, bulky heptyl group provided similar activity to that presented by bromo-substituted **4f**. This finding indicates that there may be an important hydrophobic pocket surrounding this portion of the molecule after binding.

**Figure 1.**
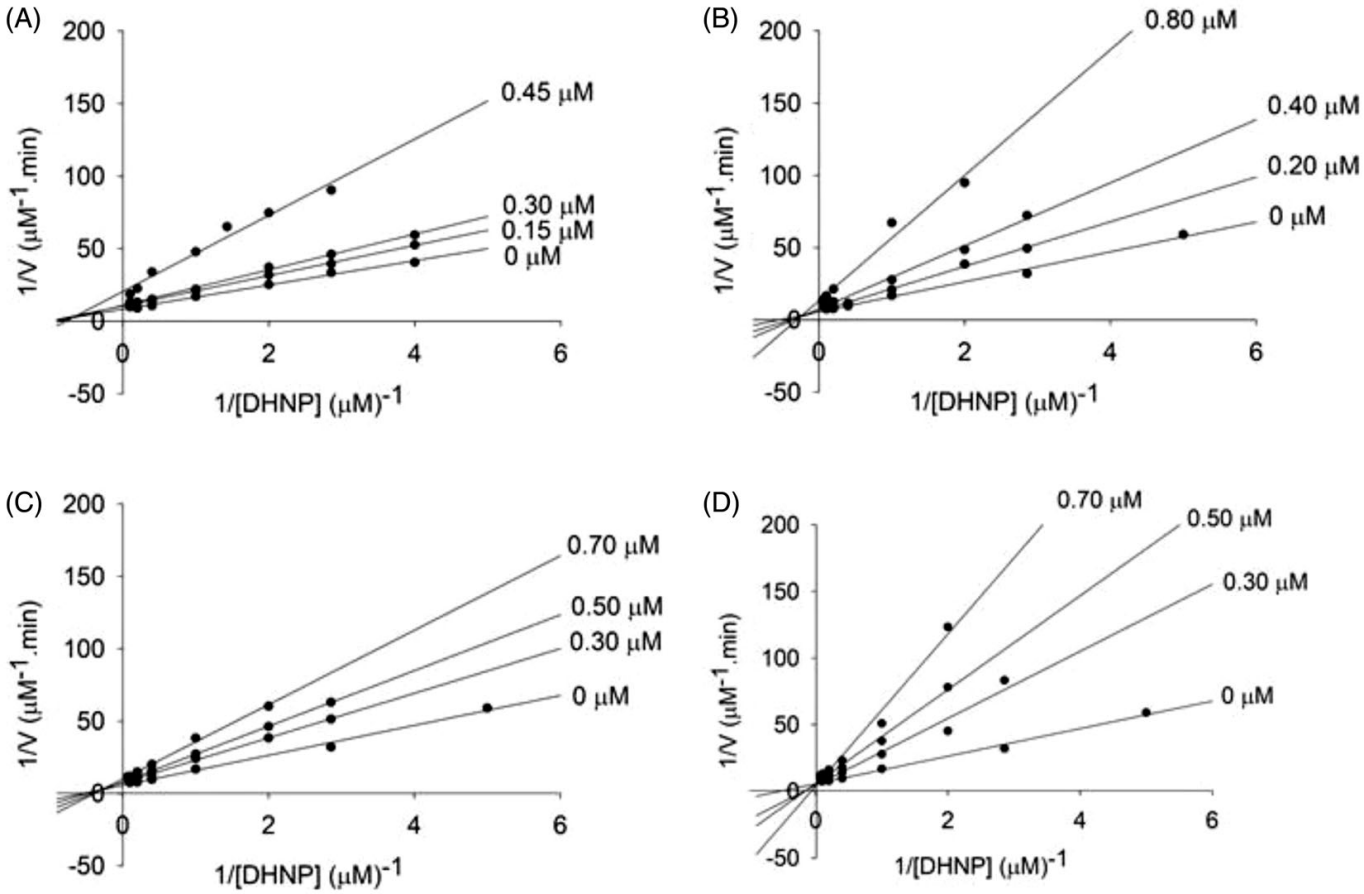
Determination of the inhibition mechanism for compounds 8-MG (**1**), **3 b**, **4f** and **4 h** on aldolase activity of *Mt*FolB. (A) Compound 8-MG (**1**) (0–0.45 µM). The Lineweaver − Burk plot displays a pattern of intersection to the left of the *y*-axis towards DHNP, which is diagnostic of non-competitive inhibition. (B) Compound **3 b** (0–0.80 µM). The Lineweaver − Burk plot displays a pattern of intersection to the left of the *y*-axis towards DHNP, which is diagnostic of non-competitive inhibition. (C) Compound **4 h** (0–0.70 µM). The Lineweaver − Burk plot displays a pattern of intersection to the left of the *y*-axis towards DHNP, which is diagnostic of non-competitive inhibition. (D) Compound **4f** (0–0.70 µM). The Lineweaver − Burk plot displays a pattern of intersection at the *y*-axis, which indicates competitive inhibition.

Using an IC_50_ value < 0.50 µM as threshold, the mode of inhibition of four compounds (8-MG (**1**), **3b**, **4f** and **4h**) was determined from Lineweaver − Burk plots. The data was fitted to the appropriate equations to give values for the inhibition constants (*K*_is_ and/or *K*_ii_)^16^ (Table 1). For 8-MG (**1**), **3b** and **4h**, the double-reciprocal plots resulted in a set of lines that intercept on the left of the *y*-axis (Figure 1(A–C)), indicating a non-competitive inhibition mode. The *in vitro* inhibition constant values *K*_ii_ and *K*_is_ for these compounds were determined fitting to Equation (3), where *K*_is_ ranged from 0.3 − 0.5 µM and *K*_ii_ ranged from 0.6 − 1.2 µM. This analysis was consistent with a typical effect of a non-competitive inhibitor with *K*_is_ < *K*_ii_. Therefore, the inhibitory profile suggests that these three compounds inhibit both the free enzyme and the enzyme − DHNP binary complex, being more effective inhibitors towards the free enzyme^16^. For **4f**, the double-reciprocal plots resulted in a set of lines that intercept at the *y-*axis (Figure 1(D)), indicating a competitive inhibition mode. This inhibitor binds to the free enzyme, disrupting substrate binding^16^. Importantly, compound **4f** was found to have a lower *K*_is_ value (0.1 ± 0.03 µM) than 8-MG (**1**) (0.3 ± 0.1 µM) (see Table 1), indicating that this derivative is a more potent inhibitor than the scaffold molecule **1** (8-MG).

**Table 1.** Inhibitory constants of selected compounds on *Mt*FolB activity.

Compound	*K*_is_ (µM)	*K*_ii_ (µM)	Inhibition mode
**1 (8-MG)**	0.3 ± 0.1	0.6 ± 0.1	Non-competitive
**3b**	0.4 ± 0.1	0.9 ± 0.2	Non-competitive
**4f**	0.1 ± 0.03	–	Competitive
**4h**	0.5 ± 0.1	1.2 ± 0.2	Non-competitive

**Figure 2.**
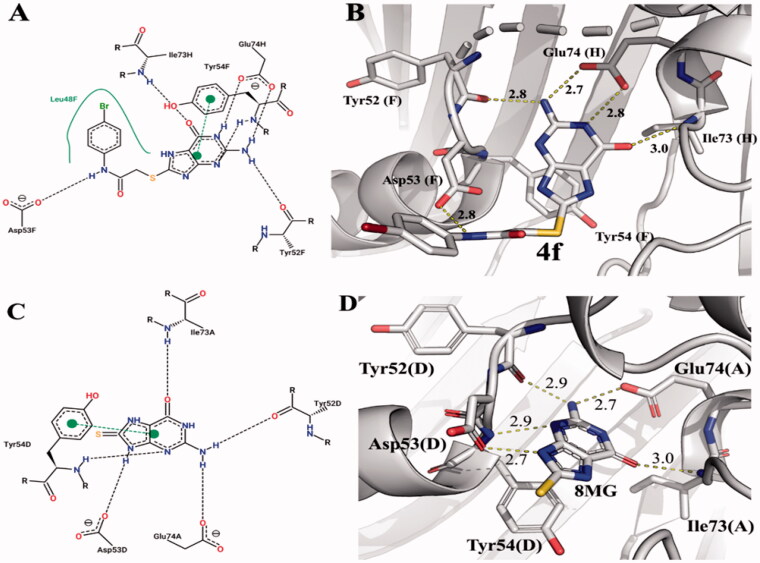
Predicted binding mode of compounds **4f** (A,B) and 8-MG (**1**) (C,D) into the binding pocket of *Mt*FolB. (A,C) 2 D-interaction diagrams of the binding models of **4f** (A) and 8-MG (**1**) (C) with *Mt*FolB residues, with hydrogen bonds and π–π stacking interactions shown in dashed lines. (B and D) Predicted docking orientations of **4f** (B) and 8-MG (**1**) (D) into the binding pocket of *Mt*FolB (PDB ID: 1NBU).

The interaction modes of compound 8-MG (**1**) and its derivatives at the active site of *Mt*FolB were evaluated using molecular docking studies. The predicted stabilities of the octameric form of *Mt*FolB bound to inhibitors were determined for 8-MG (**1**) and all derivatizations by docking simulations (Table S1). These data are presented together with IC_50_ values determined in this study. From the four compounds with IC_50_ values equal or lower than 0.5 µM, compound **4f** is the most potent inhibitor, with a k_i_ of 0.1 ± 0.03 µM (Table 1). It is indeed the only derivatized compound found to be a more potent inhibitor than compound 8-MG (**1**) (k_is_: 0.3 ± 0.1 and k_ii_: 0.6 ± 0.1 – Table 1) and the only one to display a competitive inhibition mode (Figure 1). The predicted interactions of both 8-MG (**1**) and compound **4f** with *Mt*FolB active site were compared (Figure 2). According to our results from the docking simulations, the inhibitors are associated to the binding pocket mainly by hydrogen bonds, π–π stacking, and hydrophobic interactions. In the compound 8-MG (**1)**, the amino group attached at 2-position of dihydro-purinone ring established hydrogen bonds with Tyr52D and Glu74A at distances of 2.9 and 2.7 Å, respectively. Similar distances were observed in another two hydrogen bonds involving the 3-N and 9-NH with Tyr54D and Asp53D residues. While the NH group was positioned at a distance of 2.7 Å from Asp53D, the 3-N formed a hydrogen bonding donor-acceptor pair with a distance of 2.9 Å from Tyr54D. The complex formed between compound 8-MG (**1**) and *Mt*FolB was also stabilised by π–π stacking interactions between phenyl group of the Tyr54D and the dihydro-purinone ring.

Following the same pattern presented by compound 8-MG (**1**), the main contacts between dihydro-purinone **4f** and *Mt*FolB were performed by the heterocyclic ring. The amino group (2-NH_2_) acted as a hydrogen bonding donor with distances of 2.7 and 2.8 Å from Glu74H and Tyr52F, respectively. The Glu74H carboxylate performed another hydrogen bond with the NH of the 1-position of heterocycle at distance of 2.8 Å. Unlike observed in the binary complex of 8-MG (**1**)-*Mt*FolB, the carboxylate group of Glu74H showed both oxygen atoms in a complete interaction with **4f**, fact that provides greater stability to the system. The nitrogen at 3-position of the heterocyclic ring showed distance and angle consistent with the formation of a hydrogen bond with the Tyr54F backbone. The carbonyl group present in the dihydro-purinone ring acted as a hydrogen bond acceptor at distance of 3.0 Å from the Ile73H. Finally, the heterocycle was also stabilised by contacts involving the π-electron systems of the Tyr54F and dihydro-purinone scaffold with the centroids positioned at distances of 4.1 Å. It is important to mention that the increased stability of the protein-ligand complex occurs when new intermolecular interactions are added. Moreover, exploring regions adjacent to the interaction site looking for new pockets to correct positioning of chemical groups can lead to new and important interactions with the molecular target. In this context, the acetamide portion of compound **4f** performed interactions via hydrogen bond with the Asp53F residue at distance of 2.8 Å. In addition, the 4-bromophenyl group was found to undergo hydrophobic interactions with the Leu48F side chain. These additional interactions may be related to the greater inhibitory activity and the distinct inhibition mechanism of **4f** as compared to 8-MG (**1**).

**Figure 3.**
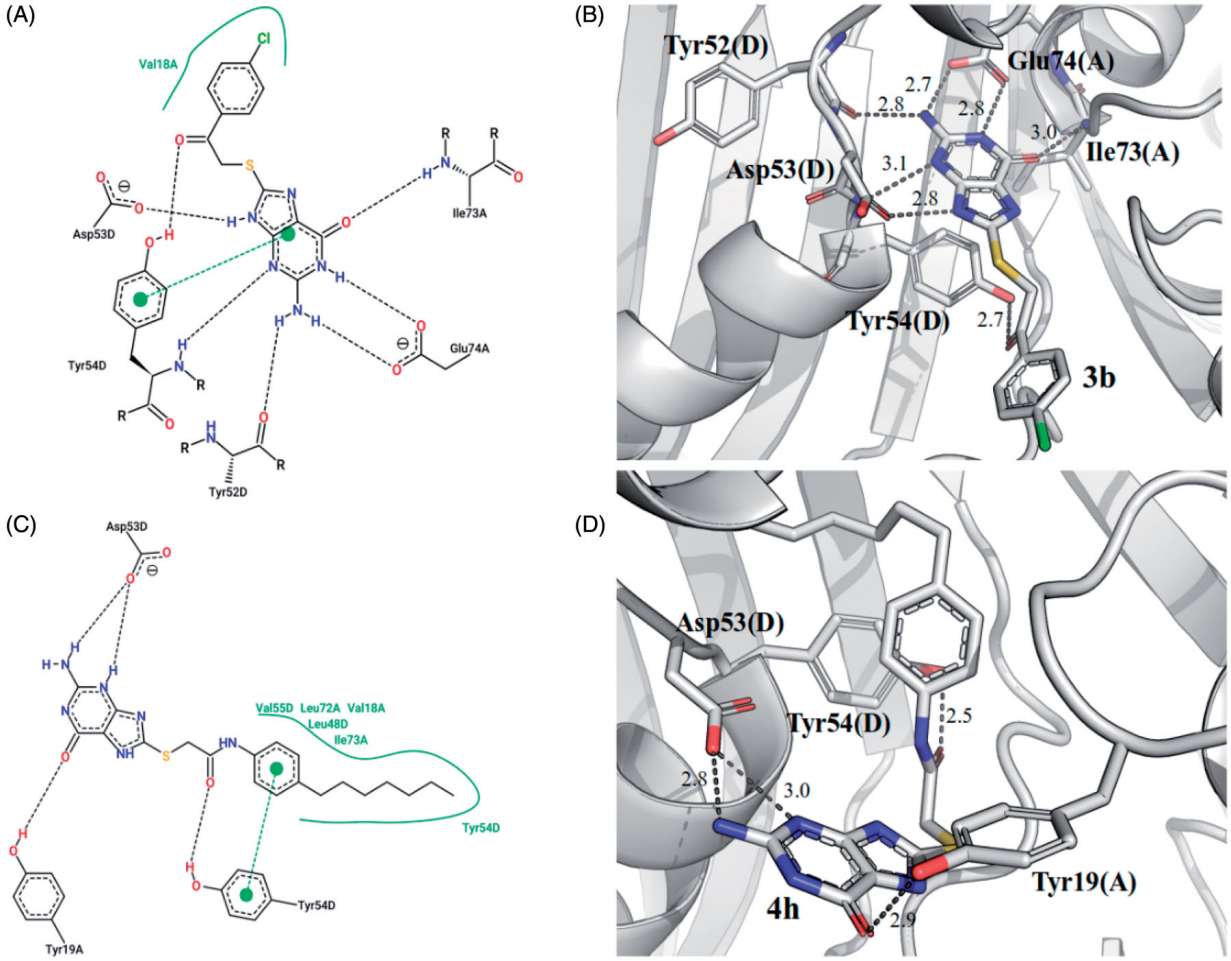
Predicted binding mode of compounds **3 b** (A,B) and **4 h** (C,D) into the binding pocket of *Mt*FolB. (A,C) 2 D-interaction diagrams of the binding models of **3 b** (A) and **4 h** (C) with *Mt*FolB residues, with hydrogen bonds and π–π stacking interactions shown in dashed lines. (B and D) Predicted docking orientations of **3 b** (B) and **4 h** (D) into the binding pocket of *Mt*FolB (PDB ID: 1NBU).

Structural poses for the non-competitive inhibitors **3b** and **4h** were also obtained from docking simulations (Figure 3). Compound **3b** was found to share many of the interactions identified in both compounds **4f** and 8-MG (**1**). Similar to compounds **4f** and 8-MG (**1**), the amino group at 2-position of its dihydro-purinone ring was found to undergo hydrogen bonds with both Tyr52D and Glu74A at equivalent distances (2.8 Å and 2.7 Å, respectively). The residue Ile73A also establishes a hydrogen bond with the carbonyl group of the dihydro-purinone ring, at the same distance of 3.0 Å found in both compounds **4f** and 8-MG (**1**). Moreover, the Tyr54 residue establishes both π–π stacking interactions and hydrogen bonds with the dihydro-purinone ring of the three compounds. A hydrogen bond of Glu74A with 1-NH of the dihydro-purinone ring (2.8 Å) also present for compound **4f** (but not 8-MG (**1**)) and another hydrogen bond of Asp53D with 9-NH of the same ring (2.8 Å) shared with compound 8-MG (**1**) (but not **4f**) completes the set of interactions shared among these compounds. Finally, the 4-chlorophenyl portion of compound **3b** establishes hydrophobic interactions with Val18A residue.

The structural pose of compound **4h** bound to the octameric form of *Mt*FolB reveals a completely different binding mechanism. None of the interactions described above shared by compounds 8-MG (**1**), **4f** and **3b** within the binding pocket of *Mt*FolB are found in the case of compound **4h**. In fact, this compound is bound in an inverted orientation relative to the others (Figure 3(C,D)). The bulky heptyl group attached at 4-position of the benzene ring in the derivatized portion of this molecule undergo extensive hydrophobic interactions with six different amino acids (Val55D, Leu72A, Val18A, Leu48D, Ile73A and Tyr54D). The Tyr54D is also establishing π–π stacking interactions with the ligand, but in this case with the benzene ring, and not with the dihydro-purinone group, as with compounds 8-MG (**1**), **4f** and **3b**. Due to its change in orientation, the dihydro-purinone group of **4h** is not found with the same interactions shared by the other three compounds. It is hydrogen bonded with only Asp53D and Tyr19A.

Interestingly, *Mt*FolB octamer bound to compound **4h** has the worst predicted free energy of binding for the derivatizations (–7.14 kcal/mol – Table S1) and experimentally was found to be the less potent of the four compounds whose mode of inhibition was investigated (*k*_is_: 0.5 ± 0.1 µM; *k*_ii_: 1.2 ± 0.2 µM – Table 1). The different modes of binding obtained from docking simulations described above could be a structural explanation for the predicted reduced stability of the inhibitor-protein complex which in turn could result in a weaker inhibition, as observed experimentally.

To evaluate whether the compounds inhibit mycobacterial growth *in vitro*, we performed a REMA experiment against the virulent Mtb H37Rv strain. The compounds presented no activity against Mtb cells (MIC values above the maximum concentration tested for each compound). Further studies will be required to evaluate the reasons for this lack of antimycobacterial activity in REMA experiments. Nevertheless, this study represents the first step towards the development of new drugs targeting FolB enzyme from *M. tuberculosis*.

## Supplementary Material

Supplemental Material
