# Supplementary material for: 8-Mercaptoguanine-based inhibitors of Mycobacterium tuberculosis dihydroneopterin aldolase: synthesis, in vitro inhibition and docking studies
Source: J Enzyme Inhib Med Chem. 2021 Mar 23;36(1):847–55. doi: 10.1080/14756366.2021.1900157 (PMC7993393; doi:10.1080/14756366.2021.1900157)
Supplement: Supplemental Material [file IENZ_A_1900157_SM0778.pdf]

Supplementary Tables

Table S1.

Compound	Experimental (µM)	Octamer (kcal/mol)
**8MG (1)**	**0.3 ± 0.1**	**-8,44**
2a	7.1 ± 1.2	-8,73
2b	1.3 ± 0.4	-9,82
2c	8.0 ± 1.3	-8,87
2d	8.0 ± 0.9	-8,79
2e	4.2 ± 1.0	-9,16
2f	2.2 ± 0.4	-9,28
2g	5.5 ± 0.7	-9,19
2h	3.0 ± 0.5	-9,44
**3b**	**0.3 ± 0.1**	**-9,47**
3c	1.3 ± 0.4	-9,14
4a	2.2 ± 0.3	-9,29
4b	0.7 ± 0.1	-8,74
4c	12.3 ± 1.8	-9,25
4d	2.0 ± 0.3	-8,74
4e	0.8 ± 0.1	-8,65
**4f**	**0.5 ± 0.1**	**-8,59**
4g	0.9 ± 0.1	-8,15
**4h**	**0.4 ± 0.1**	**-7,14**

Supplementary Figures

Figure S1

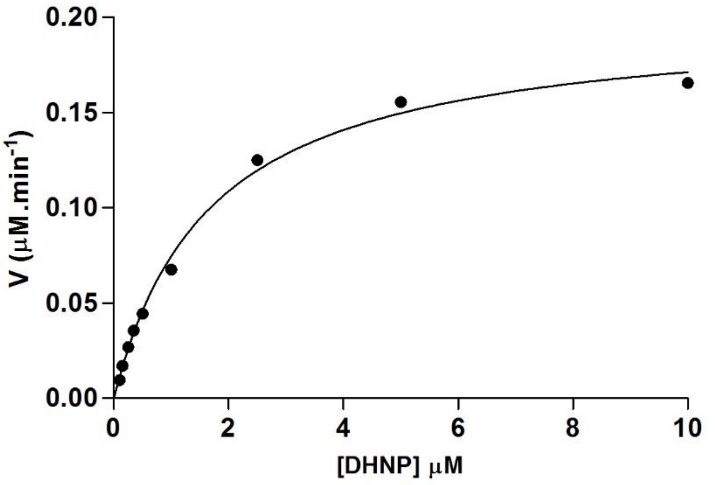

Figure S1. Determination of Michaelis–Menten constant (K_M) to DHNP substrate.

^{1}H NMR and HRMS spectra of selected compounds (**3b**, **4f**, and **4h**):

Figure S2

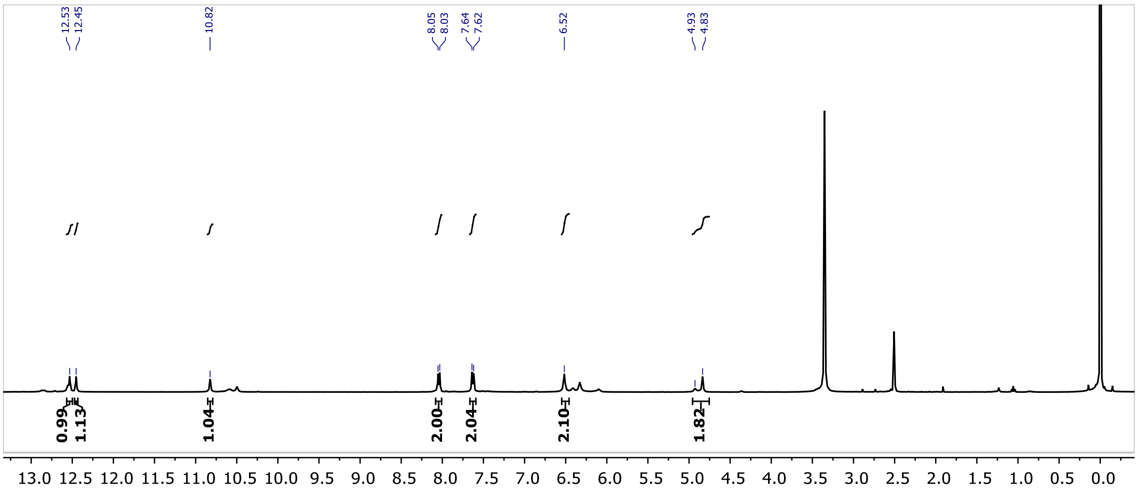

Figure S2. ^{1}H NMR spectrum of compound **3b**.

Figure S3

(a)

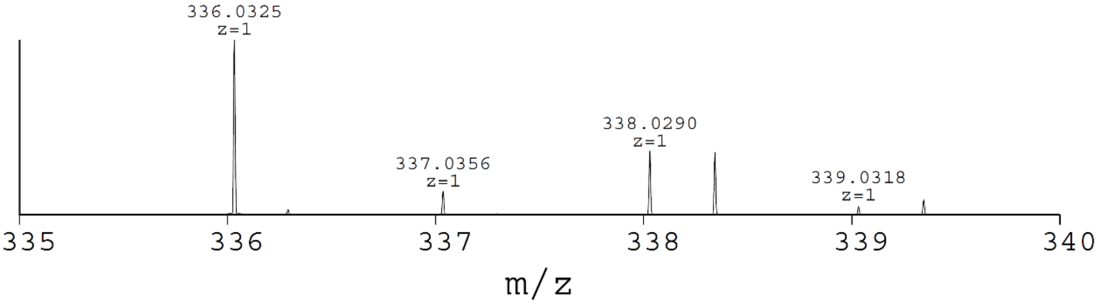

(b)

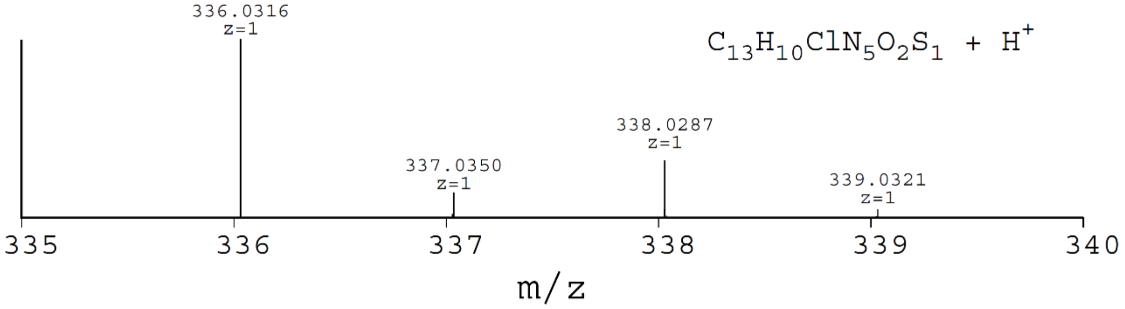

Figure S3. (a) FTMS spectra of compound **3b** obtained by electrospray ionization (ESI) in positive ion mode. **(b)** Theoretical spectra of protonated compound **3b** ($C_{13}H_{10}ClN_5O_2S_1$ + H^+).

Figure S4

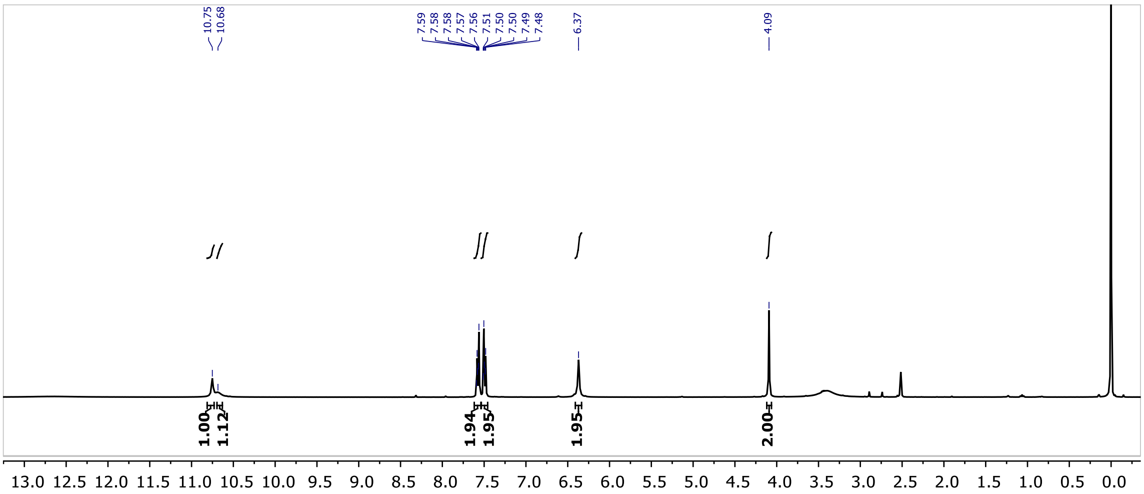

Figure S4. ^{1}H NMR spectrum of compound **4f**.

Figure S5

(a)

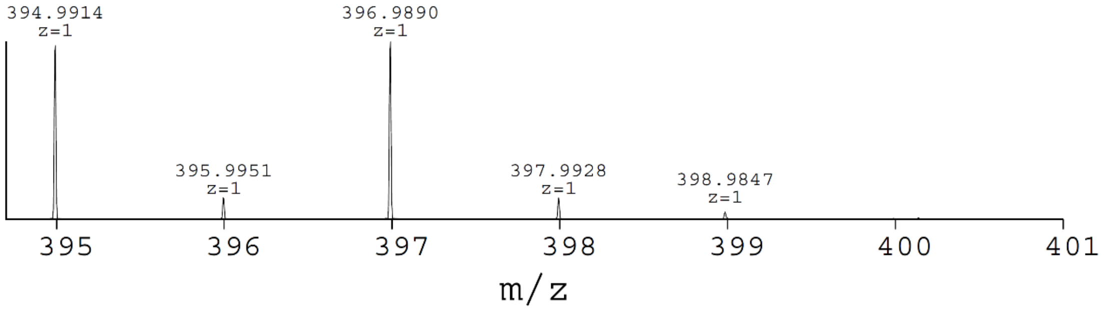

(b)

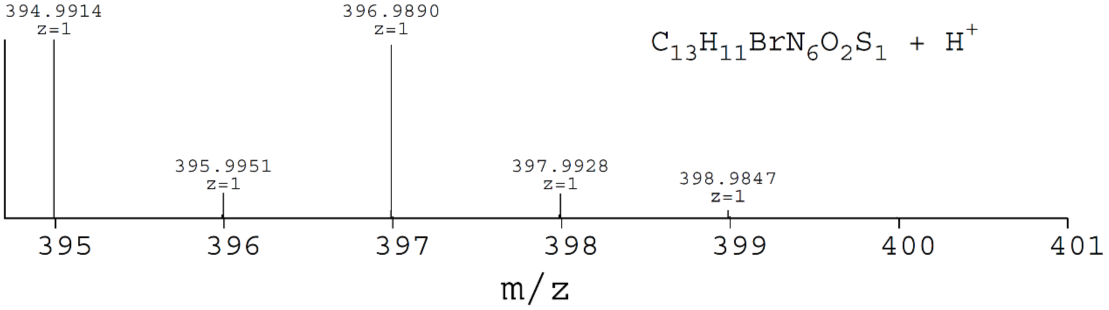

Figure S5. (a) FTMS spectra of compound **4f** obtained by electrospray ionization (ESI) in positive ion mode. **(b)** Theoretical spectra of protonated compound **4f** ($C_{13}H_{11}BrN_6O_2S_1$ + H^+).

Figure S6

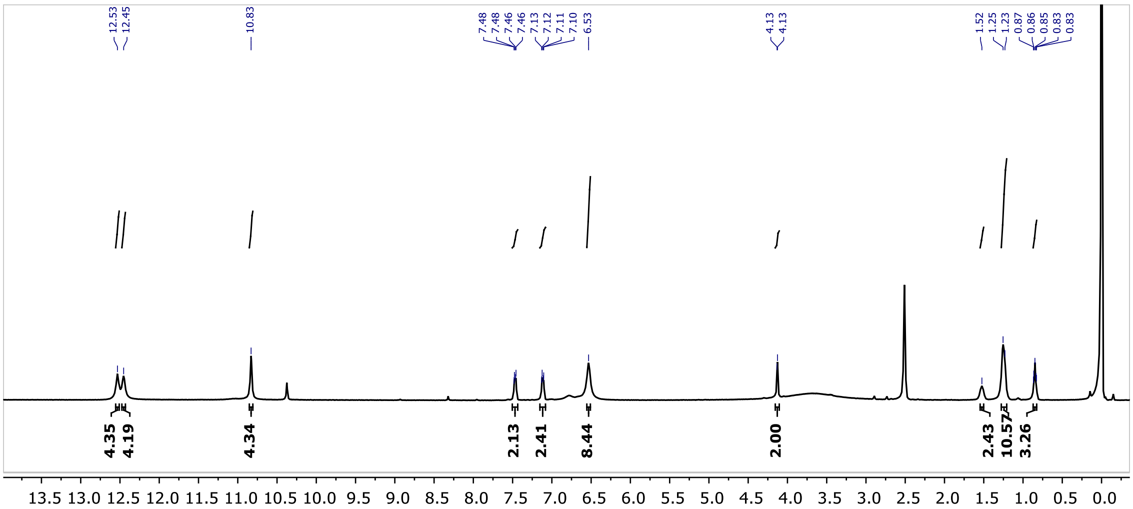

Figure S6. ^{1}H NMR spectrum of compound **4h**.

Figure S7

(a)

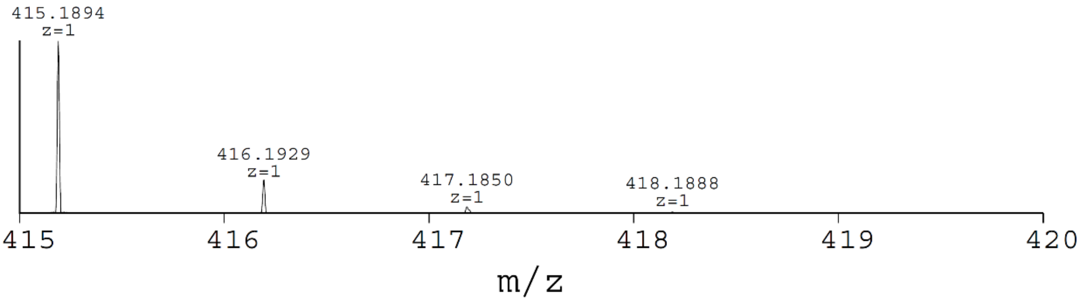

(b)

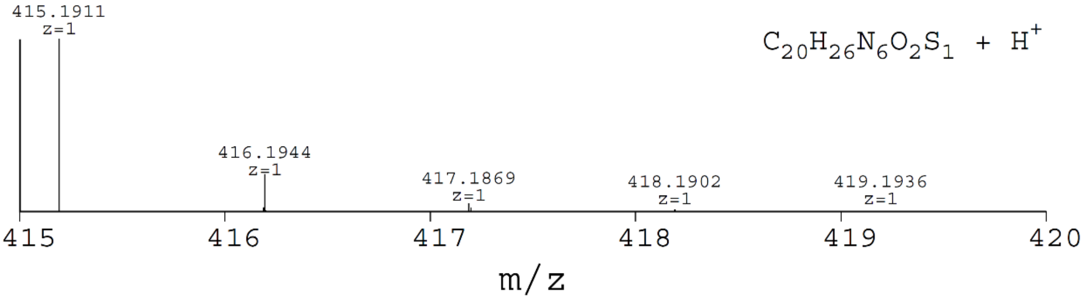

Figure S7. (a) FTMS spectra of compound **4h** obtained by electrospray ionization (ESI) in positive ion mode. **(b)** Theoretical spectra of protonated compound **4h** ($C_{13}H_{11}BrN_6O_2S_1$ + H^+).
